# Infant gut microbiota colonization: influence of prenatal and postnatal factors, focusing on diet

**DOI:** 10.3389/fmicb.2023.1236254

**Published:** 2023-08-22

**Authors:** Clara Suárez-Martínez, Marina Santaella-Pascual, Genoveva Yagüe-Guirao, Carmen Martínez-Graciá

**Affiliations:** ^1^Food Science and Nutrition Department, Veterinary Faculty, Regional Campus of International Excellence Campus Mare Nostrum, University of Murcia, Murcia, Spain; ^2^Biomedical Research Institute of Murcia (IMIB-Arrixaca), Murcia, Spain; ^3^Microbiology Service, Virgen de La Arrixaca University Hospital, Murcia, Spain

**Keywords:** gut microbiota, breast-feeding, diet, delivery mode, colonization, pregnancy, infant, newborn

## Abstract

Maternal microbiota forms the first infant gut microbial inoculum, and perinatal factors (diet and use of antibiotics during pregnancy) and/or neonatal factors, like intra partum antibiotics, gestational age and mode of delivery, may influence microbial colonization. After birth, when the principal colonization occurs, the microbial diversity increases and converges toward a stable adult-like microbiota by the end of the first 3–5 years of life. However, during the early life, gut microbiota can be disrupted by other postnatal factors like mode of infant feeding, antibiotic usage, and various environmental factors generating a state of dysbiosis. Gut dysbiosis have been reported to increase the risk of necrotizing enterocolitis and some chronic diseases later in life, such as obesity, diabetes, cancer, allergies, and asthma. Therefore, understanding the impact of a correct maternal-to-infant microbial transfer and a good infant early colonization and maturation throughout life would reduce the risk of disease in early and late life. This paper reviews the published evidence on early-life gut microbiota development, as well as the different factors influencing its evolution before, at, and after birth, focusing on diet and nutrition during pregnancy and in the first months of life.

## 1. Introduction

The human body harbors trillions of microorganisms whose coordinated actions are believed to be important for human life. Such microbial cell populations reach their highest density in the gut compartment, where they collectively form a complex microbial community known as the gut microbiota, which develops over the first 3 years of life until it reaches its adult form (Milani et al., 2017). The gut microbiota is composed of a significant number of different bacteria, approximately 160 species per person per fecal sample, and this ecosystem plays an important role in human health (Rodríguez et al., 2015). Gut microbiota members may belong to any of the three domains of life, i.e., Archea, Bacteria, and Eucarya, and also includes viruses. The gut microbiota is dominated by two phyla, Bacteroidetes and Firmicutes, followed by Actinobacteria, Proteobacteria, Verrucomicrobia, Fusobacteria, and Cyanobacteria in lower proportions (González-Bermúdez et al., 2018). Bacteroidetes, which account for 90% of the gut microbiota, include bacteria belonging to the genus Bacteroides and Prevotella. Firmicutes is constituted by a large number of genera, the most important being Lactobacillus and Clostridium, and Proteobacteria contains the genera Enterobacteriaceae. The main genus belonging to the Actinobacteria phylum in the human intestine is Bifidobacterium (Sebastián-Domingo and Sánchez-Sánchez, 2018). These microorganisms are known to establish complex trophic relationships with each other and their human host, ranging from symbiosis to parasitism (Milani et al., 2017).

In general, during childhood, various behaviors of the infant such as skin-to-skin contact with the mother, the introduction of objects, as well as parts of the body in the mouth like hands and foots, especially during the stage of crawling, are actions that promote the exposure to microbes (Arrieta et al., 2014). In addition to these actions, there are some windows of opportunity for microbiota modulation, from pregnancy to childhood, where the microbiota is more affected by environmental factors.

Until now, it was believed that during the first year of life, the infant gastrointestinal microbiota should evolve from being almost sterile, as the “sterile uterus paradigm” proposes, to a complex and varied community, with its composition and structure subjected to great variability (Funkhouser and Bordenstein, 2013). However, recent studies suggest that infants host an initial microbiome from the mother through the amniotic fluid (DiGiulio, 2012; Wang et al., 2013; Collado et al., 2016; Rehbinder et al., 2018), umbilical cord blood (Jiménez et al., 2005; Wang et al., 2013) and fetal membranes (Jones et al., 2009) and receive a maternal microbe supplementation through birth and breastfeeding. As a result, for the process of transmission and when it takes place, there are currently different theories among the scientific community, but there is a certain consensus on the main factors that can influence the transmission and subsequent colonization of the intestinal microbiota of the infant, with maternal diet during gestation, use of antibiotics including intrapartum antibiotics and type of delivery being the three main influential prenatal factors (Biasucci et al., 2008; Huurre et al., 2008; Mueller et al., 2015a; Kuperman and Koren, 2016; Mirpuri, 2020; Taylor et al., 2023).

The gestation period is one of these critical windows and focusing on diet, maternal diet has been considered a key factor that could play an important role in the colonization process in several ways. One of these is because newborns are colonized with vaginal and rectal strains from the mother transiently during delivery; this maternal microbiota could be affected by the dietary pattern during pregnancy defining the maternal microbiota and therefore the colonization of the newborn (Jiménez et al., 2005).

For example, it has been shown that maternal diet and fetal immune development are related through changes in the mother’s gut microbiota (Kaplan et al., 2011; Li et al., 2014; Boutin and Finlay, 2016; Chu et al., 2016a; Mirpuri, 2020). Dietary changes during pregnancy may affect the microbial composition and diversity of the pregnant gut, as well as the production of short-chain fatty acids (SCFA). SCFA, propionate, acetate and butyrate, are the main metabolites formed by gut bacteria through the microbial fermentation of microbiota-accessible carbohydrates (MAC) and are the main source of energy for colon cells (Alsharairi, 2020a). Among many of their functions, these metabolites affect T lymphocytes and dendritic cells by binding to protein-coupled receptors and directly inhibiting histone deacetylases, which promotes the differentiation of T-helper cells (Th1, Th2; Stiemsma and Turvey, 2017; Panzer and Lynch, 2015). A diet low in MAC has been shown to reduce bacterial diversity and SCFA production, which may interfere with regulatory T-cell function and lead to inhibition of immunoglobulin A and G (IgA, IgG) production (Alsharairi, 2020a). Also, data from animal models suggests that gut microbiome transferred through the placenta produces several metabolites that act as important mediators in the development of fetal immunity (Gray et al., 2017). Therefore, understanding how the whole process of transmission, colonization, and microbial evolution takes place during the first months of neonatal life is crucial.

Another route by which maternal diet during pregnancy can lead to changes in the microbiota of the infant is through breast milk. Several authors have observed variations in breast milk microbiota and other important bioactive compounds due to the diet of the mother during pregnancy (review by Taylor et al., 2023). This microbiota, present in breast milk, will be another source of colonization during the infant’s early life and may therefore influence the colonization patterns and establishment of the infant’s gut microbiota.

To date, there are published studies that attempt to gather information on how the various factors mentioned above influence the gut microbiota of the infant (Funkhouser and Bordenstein, 2013; Milani et al., 2017; Chong et al., 2018; Selma-Royo et al., 2019). However, publications focusing on diet (García-Mantrana et al., 2016; Chu et al., 2016a; Maher et al., 2020; Mirpuri, 2020; Miko et al., 2022; Taylor et al., 2023) have not covered the prenatal and postnatal period together and have not considered other modulatory factors that play an important role in the colonization process and development of the infant gut microbiota. All these aspects have been included in this review.

The present review is intended as a synopsis summarizing the different research and articles that focus on all these factors, with emphasis on diet. A critical appraisal of other research has been made with the aim to put them in context in an orderly, precise, and analytical manner while pointing out the similarities and differences in the literature reviewed.

## 2. Methods

A literature search was performed using the electronic databases PubMed/Medline and Scopus with no date limits, for the description of the “theory of colonization in the uterus” in a timeline format, and with date limits (from January 1, 2000 to July 5, 2023) for the analysis of prenatal and postnatal factors that influence the intestinal microbiota of the infant. The most relevant published studies were identified in an independently way by the authors.

The keywords used were (alone or in combination): colonization, meconium, amniotic fluid, uterus, sterile uterus paradigm, intestinal microbiota, pregnancy, lactation, diet, perinatal/postnatal, factors, antibiotics, gestational age, delivery, geographical, siblings, lifestyle, probiotics, dietary pattern, breastmilk, infant formula, weaning, complementary feeding, vegetarian diet, high fat diet, ketogenic diet, gluten-free diet, and infant gut microbiota. The search included reviews/systematic reviews, meta-analyses, randomized controlled trials/experimental studies, and observational studies (case, cross-sectional, case–control, cohort reports) published in English.

## 3. Transmission, colonization and evolution of the infant gut microbiota

### 3.1. Prenatal transmission and colonization: “*The sterile womb paradigm*” vs. “*in-utero* colonization hypothesis”

Until recently, the *in-utero* environment has been considered sterile under normal conditions, and the colonization process was thought to begin at birth when the infant is exposed to the microbiota of the mother and the environment (Koleva et al., 2015). According to this concept, microbes are acquired both vertically (from the mother) and horizontally (from other humans or the environment) during and after the birth (Perez-Muñoz et al., 2017).

Since the studies of Escherich (1989), who first describe the meconium (the earliest stool from an infant) to be free of viable bacteria, the idea that term fetuses are sterile *in utero* has been widely accepted (Perez-Muñoz et al., 2017). A few years later, Tissier (1900) established that bacterial colonization of the newborn occurs when the newborn initiates transit through the birth channel via contamination by maternal vaginal and fecal bacteria. The process continues after delivery and progressively the intestinal lumen of the newborn becomes colonized by temporal microbiota. The abundance and variety suffer changes over time to culminate into a relatively stable microbial composition seen throughout adulthood (Jiménez et al., 2008). Most of the studies that established the sterile womb paradigm employed traditional culture-based methods and microscopy, which are still considered valid today despite these may fail to detect viable but non-cultivable microbes (Milani et al., 2017).

For more than a century, the hypothesis of Escherich was generally accepted even though other three additional independent studies conducted in 1927 (Burrage, 1927), 1934 (Hall, 1934), and 1936 (Snyder, 1936), suggesting some microbial activity in meconium which supposed a conflict between both ideas.

After the early studies on the meconium discussed above, further microbiological research on this topic ceased for a period of over 60 years (Koleva et al., 2015) until Jiménez et al. (2008). They reported 100% (*n* = 21) of meconium to be positive for bacteria by culture techniques and therefore rebut the non-sterility of infant meconium shown by earlier researchers. Two years later and using non-culture-based techniques, Mshvildadze et al. (2010) provided further evidence on the presence of bacteria in meconium obtained from neonates born at 22 to 32 weeks gestational age. These authors detected microbial DNA in 91% of samples and denaturing gradient gel electrophoresis (DGGE) profiling revealed an association between prematurity and reduced meconium microbial diversity.

There is now a multitude of recent studies employing next-generation DNA sequencing techniques that have challenged the traditional view of human microbiome acquisition. These studies propose that neither the fetus, the placenta, or the amniotic fluid are sterile, and that acquisition and colonization of the human gastrointestinal tract begins *in utero*, what is known as “*in-utero* colonization hypothesis” (Jiménez et al., 2008; Aagaard et al., 2014; Collado et al., 2016).

Madan et al. (2012), in a prospective longitudinal study, applied high throughput pyrosequencing of the hypervariable V6 region of the 16S rRNA gene to understand the gut microbial colonization in prematurity; they found that meconium of all subjects (*n* = 6) was not sterile, being *Lactobacillus*, *Staphylococcus* and Enterobacteriaceae the predominant bacterial genera. Gosalbes et al. (2013) characterized meconium microbiota in 20 term newborns from a Spanish birth cohort, to assess whether it contributes to the future microbiota of the infant’s gastrointestinal tract, and to evaluate how it relates to lifestyle variables and atopic-conditions. Their conclusion was that the microbial pattern differed from the microbiota of feces, vagina, and skin from adults but was similar to that of young infant feces. Another conclusion of this study was that meconium microbiota has an intrauterine origin, which is influenced by maternal factors and may have consequences for childhood health.

A few years later, Moles et al. (2013) aimed to characterize the evolution of gut microbiota during the first 3 weeks of life of 14 preterm neonates, including the meconium microbiota. The bacterial diversity and taxonomy were examined using culture-dependent and molecular techniques, including DGGE and Human Intestinal Tract Chip (HITChip) analysis of 16S rRNA amplicons. Both approaches showed that spontaneously released meconium of such neonates contains a specific microbiota that differs from those observed in early fecal samples (Rodríguez et al., 2015). The phylum *Bacilli* and Firmicutes were the main bacteria groups detected in meconium, while Proteobacteria phylum was more abundant in fecal samples. Another study, carried out by Hu et al. (2013), aimed to assess if the bacterial community of meconium is affected by maternal diabetes status. Samples were collected from 23 newborns stratified by maternal diabetes status and the microbiome was profiled using multi-barcode 16S rRNA sequencing followed by taxonomic assignment and diversity analysis. A diversified microbiota was found in all meconium samples, which was not affected by the mode of delivery, showing a lower species diversity, higher sample-to-sample variation, enrichment of Proteobacteria and reduction of Bacteroidetes compared to adult feces. The taxonomy analyses, among the meconium samples, suggested that the overall bacterial content in meconium significantly differed by maternal diabetes status. Specifically, in those samples of the diabetes group, the phyla Bacteroidetes and the genus *Parabacteroides* were enriched. Two years later, one study by Hansen et al. (2015), showed evidences of microbial presence in 66% (10 of 15) of meconium samples, from 15 healthy full-term vaginally-delivered infants. The taxa which predominated in meconium belonged to *Bifidobacterium*, *Enterobacteriaceae*, *Enterococcaceae*, and *Bacteroides-Prevotella* (Koleva et al., 2015).

Shi et al. (2018) used a metagenomic sequencing technique to characterize the meconium microbiome from a Chinese cohort of vaginally and C-section delivered infants (CSDI); the authors founded, in contrast to Hu et al. (2013) the meconium microbiome diversity was higher in vaginally delivered infants (VDI) than that in CSDI. *Propionibacterium* species were most abundant in the VDI, whereas the CSDI group had high levels of *Bacillus licheniformis*. Moreover, different modes of delivery affected the antibiotic resistance gene prevalence, what might influence the infant’s health later in life. In this sense, Dominguez-Bello et al. (2010) also studied the impact of birth mode on bacterial communities in a rectal swab obtained at birth. They also found that the meconium microbiota composition of full-term infants (FTI) was also influenced by the mode of delivery (Koleva et al., 2015).

Furthermore, Tapiainen et al. (2018) investigated if maternal factors during pregnancy, such as the environment, influence the microbiome of the first stool more than immediate perinatal factors. Regions of the bacterial 16S rRNA gene were sequenced to characterize the microbiome of the first-pass meconium samples (*n* = 212). With a relative abundance of 44 %, Firmicutes was the most abundant phyla, Proteobacteria, 28 %, and Bacteroidetes, 15 %. The diversity of microbiome was increased by the biodiversity of the home environment, whereas perinatal factors, such as the delivery mode or exposure to antimicrobials during the birth did not have any effect.

In summary, despite the fact that culture and molecular techniques in diverse publication have provided preliminary evidences for diverse groups of bacteria in meconium from both FTI and preterm infant, its origin remains unclear. Meconium microbial communities have low species diversity and high inter-individual variability, thus being very similar to early fecal microbiota (Hu et al., 2013; Moles et al., 2013). At the phylum level, meconium microbiota looks more closely to gut microbes of infants than of adults (Dominguez-Bello et al., 2010). Despite this similarity, when compared meconium with fecal samples at 3 and 12 months, meconium was found to be less abundant in *Bacteroides* and *Bifidobacterium* species and therefore more likely to be colonized with *Escherichia-Shigella* and *Enterococcus* (Bäckhed et al., 2015). Moreover, the high similarity between meconium and amniotic fluid microbes (Ardissone et al., 2014), and the fact that large quantities of amniotic fluid are swallowed by fetus in the last trimester of pregnancy (Gilbert and Brace, 1993), leads to the assumption that the meconium microbiota may have an intrauterine origin. As we reviewed in detail in the next section, these findings contradict the classic dogma whose main idea is that the newborn comes from a sterile environment and suggest that the establishment of intestinal microbiota is initiated in the prenatal gut (Koleva et al., 2015).

On the other hand, in two recently published papers (Dos Santos et al., 2021; Kennedy et al., 2021), the authors have compared the sequencing of meconium samples and negative controls (a swab exposed to operating theatre air during delivery, genomic prep reagents either exposed to PCR hood air during sample preparation or not exposed and PCR amplifications without added template DNA) traying to elucidate if meconium is sterile or not, and in both they observed no significant differences in the number of reads or taxonomic composition between negative controls and meconium, which again supports “*the sterile womb paradigm.”*

### 3.2. Origin of meconium microbiota and routes of transmission

Thanks to the new advances in sequencing and molecular technologies, it has been possible to study the microbiome of areas considered sterile. Some recent studies have provided evidences of the presence of microbes in placental tissue (Aagaard et al., 2014), amniotic fluid (DiGiulio, 2012; Wang et al., 2013), umbilical cord blood (Jiménez et al., 2005), fetal membranes (Jones et al., 2009) and meconium (Hu et al., 2013; Moles et al., 2013; Collado et al., 2016; Shi et al., 2018) the existence of functional pathways that allow a bacterial exposure with the fetus during the stage of pregnancy (Koleva et al., 2015).

While vaginal microbes associated with preterm birth can get access to the uterine environment through an ascending route, the mechanisms by which gut bacteria reach this human niche (placenta, amniotic fluid and umbilical cord) are not well understood.

There are still many doubts about what could be the mechanisms by which intestinal bacteria gain access to the uterine environment. It has been suggested that gut microorganisms after translocation of the gut epithelium, are able to travel to the placenta through the bloodstream. It is known that one of the main roles of the intestinal epithelial barrier is to prevent microbial entry into the circulatory system, but dendritic cells can actively penetrate the intestinal epithelium to the intestinal lumen where bacteria are present, elevate these live bacteria and transport them through the body as they migrate to the lymphoid organs (Rodríguez et al., 2015).

Furthermore, bacterial species that are normally found in the human oral cavity have also been isolated from the amniotic fluid and probably these bacteria, during periodontal infections, may have access to the bloodstream thanks to the inflammation of the gums. The main bacterial species, with an oral origin, found in amniotic fluid are *Fusobacterium nucleatum*, *Streptococcus* spp., *Bergeyella spp.*, *Porphyromonas gingivalis*, *Rothia dentocariosa*, and *Filfactor alocis* (Funkhouser and Bordenstein, 2013).

To test whether maternal gut bacteria can be transferred to fetuses *in utero*, two pioneer studies investigated if oral administration of a genetically labelled *Enterococcus faecium* to pregnant mice resulted in its presence in amniotic fluid and meconium of term off-spring after sterile c-section (CS; Jiménez et al., 2005, 2008). In both studies, *Enterococcus faecium* genetically distinctive strain was detected by polymerase chain reaction in the intestinal lumen of pups delivered 1 day prematurely by CS. Also, *E. faecium* with the genetic label was cultured from amniotic fluid and meconium of pups from inoculated mothers. In contrast, it could not be detected in the samples obtained from a non-inoculated control group.

On the other hand, other studies have concluded that there is no evidence of a placenta, amniotic fluid and/or early contact with microbes (Lauder et al., 2016; Rehbinder et al., 2018; Panzer et al., 2023) and therefore, the *in-utero* colonization hypothesis continues to be the subject of debate (Selma-Royo et al., 2019). A review published by Perez-Muñoz et al. (2017) concluded that current scientific evidence does not support the existence of microorganisms in the fetal healthy environment.

With new molecular and sequencing techniques, it has been possible to overcome the limitation of culture-based methods, but it is true that the data that support the *in-utero* colonization hypothesis must be taken with extreme caution, because of particular methodological limitations. For example, the bacterial DNA detected may belong to non-viable microorganisms, that is, dead microorganisms which cannot be detected by culture-based techniques. In the case of studies carried out in placenta this consideration is particularly important because it plays a key role in the elimination of microbes and other components that may be ubiquitous in the blood (Perez-Muñoz et al., 2017; Panzer et al., 2023). Another important methodological issue is that the highly sensitive molecular techniques employed to study the low microbial biomass, may detect contaminating microbes and therefore produce false-positive results (Milani et al., 2017).

## 4. Microbial colonization and their evolution during the first months of life: how the establishment of the microbiota occurs?

It is from birth when the human microbial colonization process begins to a greater extent and continues to develop and modulate in species abundance for about 3 years, until the microbiota becomes adult-like. A great number of trials in both animal models and humans, suggests that this period constitutes a critical window for immunological and physiological development (Arrieta et al., 2015).

For example, the intestinal-barrier plays an important role in the defense against infections, and nutritional, endocrine, and immune functions. Increased permeability may cause beneficial effects but also may enhance the uptake of microorganisms and foreign antigens, leading to risk of development of infection, inflammation and systemic hypersensitivity (Łoniewska et al., 2020). At birth, intestine is highly permeable and then drops sharply after delivery, which leads to a process known as “gut closure” during the first year of life. It has been shown that the use of antibiotics and vaginal delivery have been associated with an increase in the intestinal permeability during this period (Łoniewska et al., 2020) and that *Ruminococcus* (torques group) might be one of the microorganisms most involved in controlling paracellular permeability (Kaczmarczyk et al., 2021).

**Figure 1:**
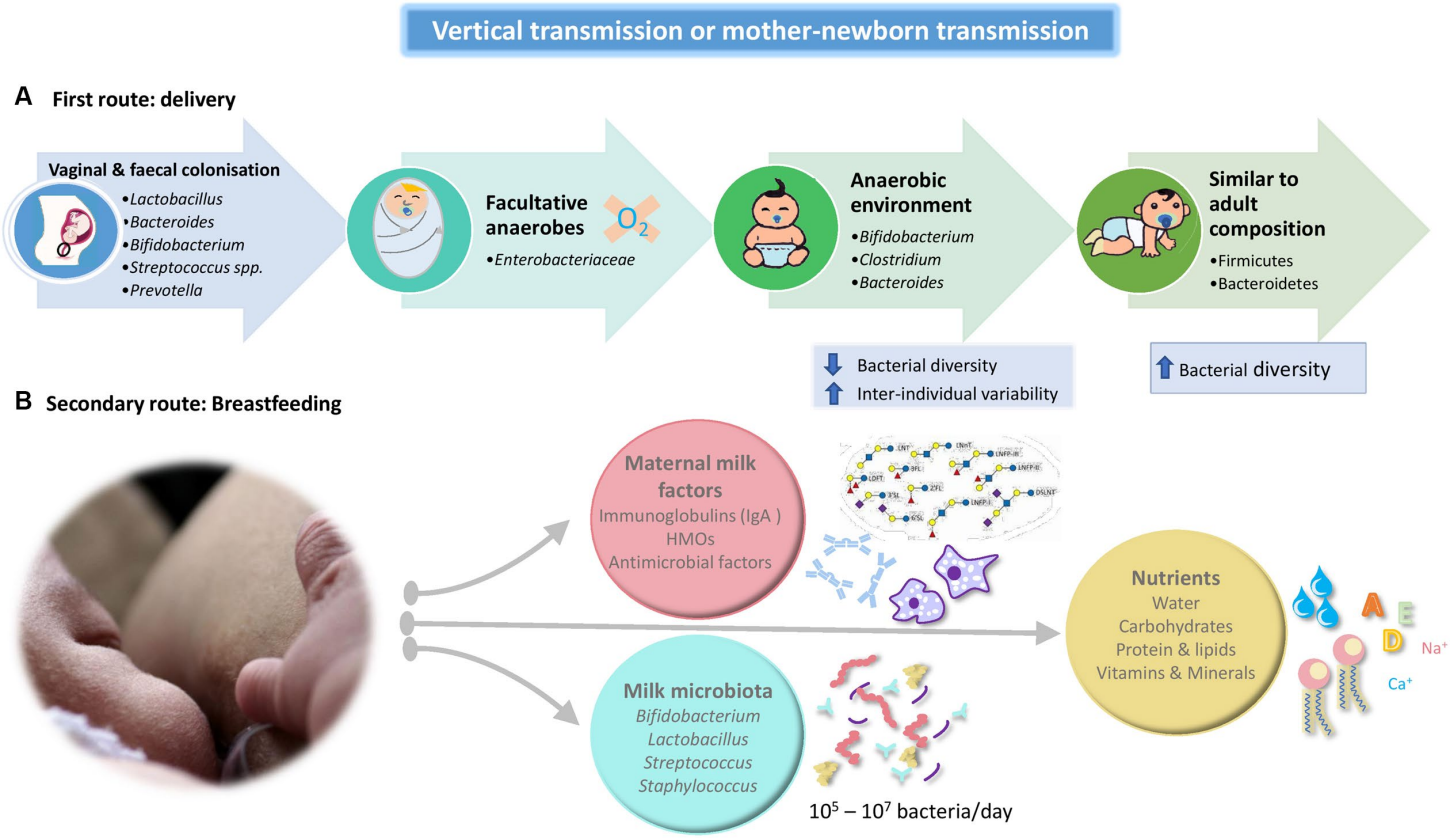
Schematic summary of the two main routes that comprise the vertical transmission or mother-newborn transmission. **(A)** Route linked to the type of delivery. **(B)** Route linked to breastfeeding. HMO: Human Milk Oligosaccharides.

The great advances in metagenomic technologies during the last 20 years have allowed us to know more accurately the composition of the intestinal microbiota since early infancy (Bäckhed et al., 2015; Hill et al., 2017; Murphy et al., 2017). Different genotype-based studies have provided definitive evidence of the transmission of specific microbes from mothers to the gut of their infants (Dominguez-Bello et al., 2010; Bäckhed et al., 2015; Asnicar et al., 2017). This concept of transmission between mother and infant is commonly known as vertical transmission or mother-newborn transmission (Figure 1). For example, Bäckhed et al. (2015), using metagenomic sequencing, compared the microbial species in 98 newborns and their mothers and found that 135 of 187 taxonomically annotated MetaOTUs present in vaginally delivered newborns also were found in their own mothers including important species such as Escherichia/Shigella, *Bifidobacterium longum*, *Enterococcus faecalis*, *Bacteroides fragilis*, *B. thetaiotaomicron*, and *Bilophila wadsworthia*, reinforcing the concept of vertical transference. Also, Dominguez-Bello et al. (2010) demonstrated that neonates born vaginally have a microbiota resembling the vaginal microbiome, enriched with *Lactobacillus* and *Prevotella* species, although other bacteria, such as the *Enterobacteriaceae* family, including *Escherichia* or *Klebsiella*, are also present (Selma-Royo et al., 2019). On the contrary, Dos Santos et al. (2023) conducted a longitudinal, prospective cohort study of 621 Canadian mother-newborn pairs and collected pre-delivery maternal vaginal swabs and infant stool samples at 10-days and 3-months of life. The aim was to evaluated the effect of maternal vaginal microbiome composition on the development of the infant stool microbiome. The authors concluded that maternal vaginal microbiome composition at delivery does not affect infant stool microbiome composition and development.

During vaginal delivery, the infant gut becomes colonized by maternal vaginal and fecal bacteria (*Lactobacillus*, *Prevotella* of the Bacteroidetes phylum, *Sneathia* of the Fusobacteria phylum; Jiménez et al., 2008). First colonizers, facultative anaerobes such as members of the *Enterobacteriacea* family, create an anaerobic environment in the intestinal lumen which allows the growth of strict anaerobes as *Bifidobacterium* spp., *Clostridium*, and *Bacteroides* (Koleva et al., 2015; Rodríguez et al., 2015). The neonatal gut microbiota is characterized by low bacterial diversity and higher inter-individual variability as well as a relative dominance of the phyla Proteobacteria and Actinobacteria (mainly comprised of *Bifidobacterium* genus). The microbiota become more diverse with the emergence and dominance of Firmicutes and Bacteroidetes as time after birth increases, resembling the adult composition and diversity approximately between 2 and 5 years of age (Yatsunenko et al., 2012; Bäckhed et al., 2015; Rodríguez et al., 2015).

A secondary route of maternal microbial transmission is breastfeeding. Breast milk provides a mix of nutrients and pro microbial and antimicrobial agents, which satisfies the nutritional requirements of the infant and confers protection against pathogens through the transmission of maternal antibodies (IgA) and other antimicrobial factors (Arrieta et al., 2014; Milani et al., 2017). Maternal microbial transmission occurs in two different ways, indirectly, via maternal milk factors that affect bacterial growth and metabolism such as human milk oligosaccharides (HMO), secretory IgA, and anti-microbial factors and, directly, by the exposure of the neonate to the milk microbiota (van den Elsen et al., 2019).

Traditionally, through the use of culture-dependent techniques the presence of microbes in human milk has been confirmed. Most bacteria isolated from breast milk belong to *Staphylococcus*, *Streptococcus*, *Lactobacillus*, and *Bifidobacterium* spp., (Gomez-Gallego et al., 2016). It has been described that an infant can consume approximately 800 ml/day of milk, leading to ingest between 10^5^ and 10^7^ bacteria per day, with human milk constituting one of the main sources of bacteria to the breastfed infant gut (Fernández et al., 2013). However, the composition of breast milk microbiota is not stable over time. Various studies have been able to determine that there is an evolution throughout the period of breastfeeding. For example, Cabrera-Rubio et al. (2012) concluded that the colostrum microbiota has a higher diversity than mature milk, being dominated by *Weissella*, *Leuconostoc* (both lactic acid bacteria), *Staphylococcus*, *Streptococcus*, and *Lactobacillus* spp. One month later, the *Staphylococcus* level is dramatically reduced, and there are higher levels of *Veillonella*, *Prevotella*, *Leptotrichia* (typical inhabitants of the oral cavity), *Lactobacillus*, *Streptococcus* spp., and increasing levels of *Bifidobacterium* and *Enterococcus* spp. These techniques have confirmed the existence of a rich and diverse breast milk microbial community. Also, Ward et al. (2013) carried on a shotgun metagenomics analysis of 10 pooled human milk samples by total DNA sequencing and reported that Proteobacteria (65 %) and Firmicutes (34 %) are the predominant phyla and *Pseudomonas* spp. (61.1%), *Staphylococcus* spp. (33.4%), and *Streptococcus* spp. (0.5%) are the predominant genera.

Some reviews summarize that human milk microbiota could derive from colonization from mother’s skin and the infant’s oral cavity during suckling because breast milk flows back into the mammary ducts, which provides a route for bacteria found in their infant’s oral cavity to enter the mammary gland. But a number of studies also support the entero-mammary pathway hypothesis, wherein bacteria from the maternal gut may reach the mammary glands via dendritic cells and macrophages (Fernández et al., 2013; Gomez-Gallego et al., 2016; Milani et al., 2017; van den Elsen et al., 2019).

In addition to shaping the composition of the microbiota, the practice of early feeding affects the metabolism of the microbiota. The microbiomes of newborns and breastfed are enriched in genes necessary for the degradation of HMO. HMO are the third largest component of breast milk and are structurally complex sugars unique to human breast milk. They are not digestible, promote the proliferation and growth of specific microorganisms, including *Bifidobacterium* and Bacteroidetes, and the metabolism of these substrates resulted in the production of lactate and short-chain fatty acids (SCFA), which in turn increased the acidity of the surrounding environment, an important factor in preventing the invasion of pathogens. For all of that, HMO are considered a type of prebiotic and exert positive effects on health (Arrieta et al., 2014; Chong et al., 2018; van den Elsen et al., 2019).

Throughout the first year of life, gut bacterial diversity and richness continue to respond rapidly to changes in the infant diet. The first introduction of infants to solid foods occurs during weaning (complimentary feeding) when infants are exposed to a much larger array of non-digestible carbohydrates than those present in breast milk or formula. The presence of new substrates can significantly alter the gut microbiota, favoring the growth of polysaccharides fermenters such as *Bacteroides*, *Clostridium*, *Ruminococcus*, and *Faecalibacterium (*Arrieta et al., 2014; Vallès et al., 2014). During weaning the alpha diversity, concept that indicates how many different species could be detected in a microbial ecosystem, increases, resulting in the replacement of Proteobacteria and Actinobacteria by Firmicutes and Bacteroidetes phyla as the dominant members of the infant microbiota (Fallani et al., 2011; Bergström et al., 2014; Laursen et al., 2016; Milani et al., 2017; van den Elsen et al., 2019).

Additionally, the introduction of solid foods also changes the metabolic function of the gut bacteria as genes involved in the degradation of sugars from breast milk are less needed and utilized. Instead, the microbiota adapts to the available energy source, and functionally matures to be able to degrade complex sugars and starch found in solid food (Bäckhed et al., 2015). By the end of the first year of life, the composition of the infant gut microbiota is more similar to the microbiota of an adult; however, a typical adult microbial profile is not established until 2-3 years of age (Yatsunenko et al., 2012). The development of such condition reaches a climax status represented by the establishment of a homoeostasis among all its members. A wide range of factors can cause shifts in this microbiota balance, thereby disrupting the gut microbiota homoeostasis and causing a state of dysbiosis. There is a controversy on the exact meaning of dysbiosis, simply because of the lack of an accurate description of a “normal” or healthy microbiota. Dysbiosis is usually associated with harmful effects and may have long-term consequences leading to disorders or diseases. The process of development and maturation of the intestinal microbiota is a dynamic and non-random process, in which both positive and negative interactions take place between the main microbial taxa (Milani et al., 2017). Therefore, more research is needed to clarify what specific components of a solid food diet play the biggest role in the development of the infant gut microbiota and how it will affect the child’s health in the long term.

## 5. Main influential factors in the microbial colonization

**Figure 2:**
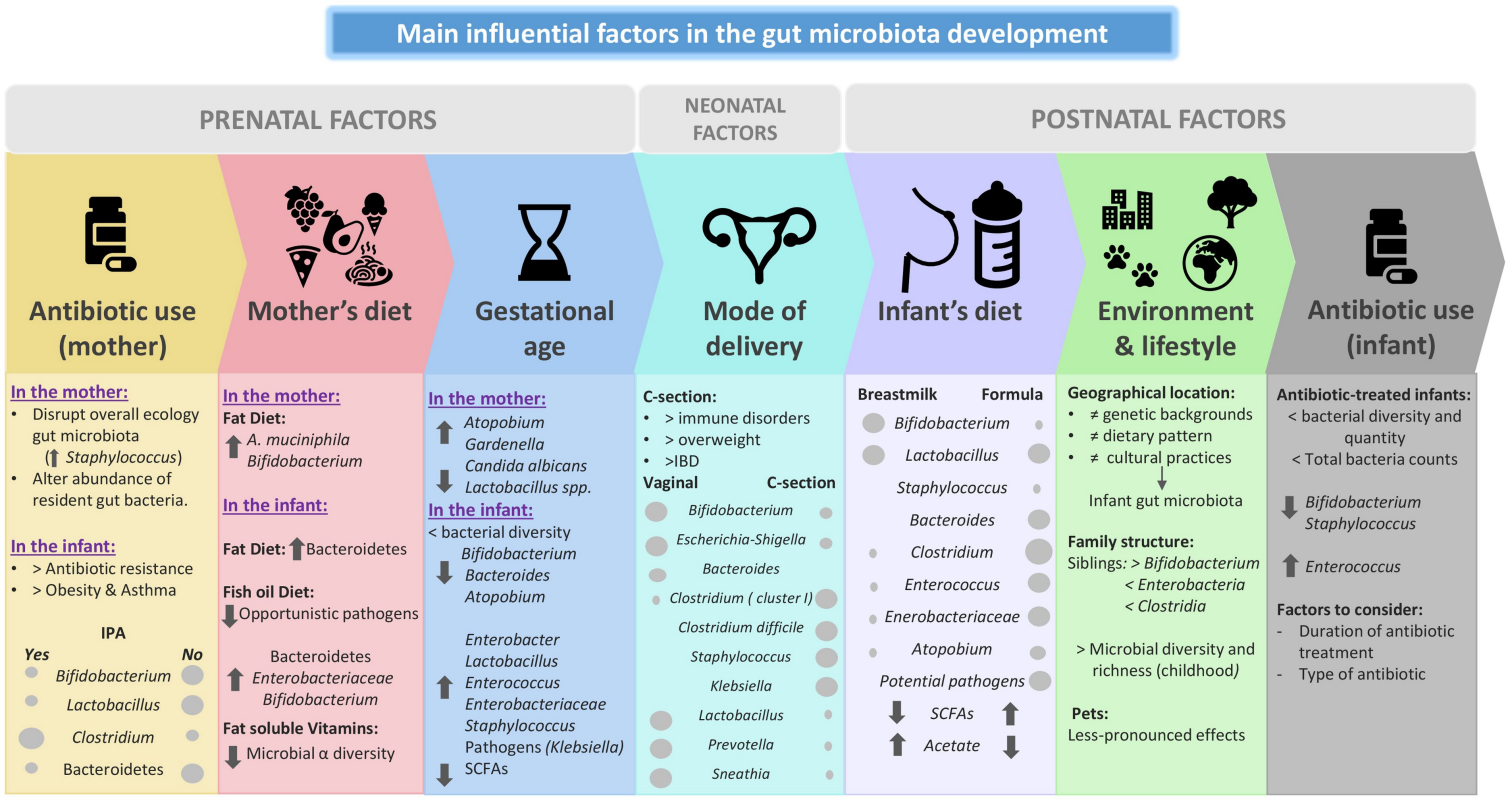
Main influential factors in the gut microbiota development. IPA: Intra partum antibiotic. SCFA: Short chain fatty acids. IBD: Infant bowel disease.

Human microbial colonization represents the *de novo* assembly of a complex microbial community, a process that is influenced by a number of different factors (both intrinsic and extrinsic) such as mode of delivery, type of feeding, and antimicrobial treatments. Also, as previously discussed, the diet during the pregnancy period and during the first years of life has an influence in this development. The mother’s age, as well as environmental and life style, and family genetics have also been reported to impact the infant microbiota (González-Bermúdez et al., 2018). Various epidemiological studies have established a clear correlation between these factors that disrupt the gut microbiota during childhood, and immune and metabolic disorders later in life (Sevelsted et al., 2015; Milani et al., 2017). The following section highlights some perinatal, neonatal and postnatal factors that are thought to influence the development of infant gut microbiota (Figure 2).

### 5.1. Prenatal and perinatal factors

Prenatal factors during pregnancy can affect the maternal gut microbiota, which in turn can affect the infant gut colonization and even influence the future development and behavior of the infant.

#### 5.1.1. Antibiotics during pregnancy

Regarding the use of antibiotics, initial epidemiological evidence indicates that disrupting microbial exchange through the use of antibiotics in pregnancy may increase offspring risk of some chronic diseases later in life, such as childhood obesity and asthma (Mueller et al., 2015b). As many reviewed resume, inappropriate antibiotic usage may significantly disrupt the overall ecology of the gut microbiota, alter the abundance of resident gut bacteria, potentially lead the child toward certain diseases, and conferring antibiotic resistance in infancy (Arrieta et al., 2014; Kumbhare et al., 2019; Dierikx et al., 2020). In one of the previous studies carried out in mice, it was observed that prenatal antibiotics decrease the diversity and structure of the microbiota (Tormo-Badia et al., 2014).

Antibiotics treatment during pregnancy is widespread in Western countries, representing 80% of the medications prescribed at this stage. However, although antibiotic treatment can sometimes save lives, it can also have harmful consequences. The use of antibiotics in the prenatal period, has been associated with delayed colonization by some microbes especially *Bifidobacterium* and *Lactobacillus* species (Jauréguy et al., 2004; Westerbeek et al., 2006; Faa et al., 2013; Keski-Nisula et al., 2013). Also, a study carried out by Mueller et al. (2015a) found that children exposed to prenatal antibiotics in the second or third trimester had 84% higher risk of obesity compared with unexposed children.

Stokholm et al. (2014) reported an increase in vaginal colonization by species of *Staphylococcus* due to the use of antibiotics during pregnancy and also observed a mayor colonization by *Escherichia coli* at women treated at the third trimester of pregnancy. All these alterations may have effects on the early microbial colonization of the neonate. However, according to more recent studies (Kamal et al., 2019; Korpela, 2021), no significant differences in gut microbiota composition were found between infants exposed and unexposed to maternal antibiotic who born by C-section, but antimicrobial alters the gut microbiota in a significantly way in vaginally delivered infants.

During the perinatal period the main cause of antibiotic exposure is the use of intrapartum antibiotic prophylaxis (IPA) in over 30 % of total deliveries (Nogacka et al., 2018). Mothers are given IPA as standard treatment for prevention of vertical transmission of Group B *Streptococcus* to neonates. This standard of care treatment is an opportunity to examine how antibiotics can affect the infant microbiome (Kim et al., 2019). It has been reported in recent studies that IPA affects the development of later microbiota in the newborn (Jauréguy et al., 2004; Keski-Nisula et al., 2013; Aloisio et al., 2014; Arboleya et al., 2015; Azad et al., 2016; Gonzalez-Perez et al., 2016). As Korpela (2021), the vaginally delivered infant’s gut microbiota pattern, whose mothers were exposed to IPA, was represented by an increase in *Clostridia, Enterobacteria, Streptococci* and pathogens species, and a decrease in *Bifidobacteria.*

More specifically, in a longitudinal prospective birth cohort, the bacterial community in early life of infants exposed to IPA were different from that infant who were not exposure at 10 days and 6 weeks of age, and these differences disappeared at 12 weeks (Stearns et al., 2017). The authors observed that Actinobacteria had a delay pattern of colonization. Also, the time of exposure to treatment influences the pattern of colonization as in this study, there was a decrease of 7.2 % in the abundance of *Bifidobacterium* and a positive effect on the abundance of *Clostridium* for each hour of IPA administration during vaginal birth. Aloisio et al. (2016) observed significant differences in the microbial composition in newborns whose mothers received IPA during delivery, with increased level of Proteobacteria and a lower number of Actinobacteria and Bacteroidetes. A similar pattern and a higher abundance of Proteobacteria and Firmicutes was also observed by Nogacka et al. (2018). These findings underscore the fact that the consumption of antibiotics by mothers during pregnancy can affect the gut microbiome of the newborn, which in turn can affect the health and development of the infant. Therefore, this factor must be carefully observed and controlled.

#### 5.1.2. Consumption of probiotics and supplements

Despite the existence of a wide range of research conducted on the use of probiotics during pregnancy and the influence on breast milk microbiota, the data on how probiotics affect the offspring’s gut microbiota are very limited. The consumption of probiotics during gestation had been related with a lower risk of atopic disease or asthma in infants (Lahtinen et al., 2009; Ismail et al., 2013).

The study carried out by Lahtinen et al. (2009) is the only research found to date that study the consumption of probiotic by the mother during gestation up to the time of delivery, and their effect in the offspring gut microbiota. The authors employed an exclusively prenatal supplementation strategy and observed an increase in the prevalence of *B. longum* among the infants (at 90 days of life) whose mothers consumed probiotics during late pregnancy and a trend towards a higher increased prevalence of *B. breve*. These results are important because these findings are in line with the entero-mammary hypothesis.

According to a recent systematic review conducted by Zaidi et al. (2021), consumption of probiotic during pregnancy and lactation was associated with the same beneficial bacteria colonization of the infant gut but with varying effects according to participant characteristics, type of supplement administered, and outcome measured. Also, this review concluded that nutritional supplementation with probiotic or vitamin D during pregnancy and lactation had very limited effects on the alpha and beta diversity of the breastmilk and infant gut microbiota (Zaidi et al., 2021). Regarding vitamin D supplementation, in the KOALA birth cohort (Talsness et al., 2017), the authors observed limited associations between maternal vitamin D supplementation, 25-hydroxyvitamin D concentration, and the infant gut microbiome. Just a negative correlation between levels of maternal vitamin D and *Bifidobacterium* spp. was also reported.

#### 5.1.3. Diet and nutrition during pregnancy

As discussed above, the maternal intestinal flora is the major source of a healthy microbiota for the newborn, and the diet during pregnancy may modify the proportion of different groups, and therefore, the pattern of colonization of the newborn *in utero* and subsequently during delivery and early life. Moreover, the changes in the mother’s microbial composition can alter the abundance of genes that promote various metabolic processes during pregnancy (Gohir et al., 2015) also affecting the microbial composition of the newborn. Several previous studies, in addition to demonstrating a clear association between the diet and the intestinal microbiome, have also successfully revealed the important role of maternal dietary modulations, since they can influence changes in the infant’s intestinal microbiome (Table 1). Moreover, it has recently been reported that the maternal diet during pregnancy has a key impact on both the maternal and infant microbiota in a delivery mode-dependent manner (Chu et al., 2016b; Lundgren et al., 2018; Selma-Royo et al., 2021).

**Table 1:** Diet and nutrition during pregnancy: description of the studies.

References	Year	Type of study	Study population	Objective	Methods	Summary of major findings
High fat diet						
Urwin et al. (2014)	2013	RCT	75 mothers and 38 infants	To investigated whether increased salmon consumption during pregnancy, maternal weight gain during pregnancy or mode of infant feeding alter the markers of gut immune defence and inflammation.	Women were randomly assigned to continue consuming their habitual diet or farmed salmon from 20 weeks of pregnancy to delivery. Faecal samples were collected from the mothers at 38 weeks of gestation and from their infants on days 7, 14, 28 and 84 post-partum. Microbiota composition by FISH.	Infants in the salmon group: ↓ *Atopobium* cluster (*p* = 0·097). This difference was significant in the FFI, but not in the EBFI.
Ma et al. (2014)	2014	CC (Primate model)	(4–9 females and 1–2 males per group)	To examine the role of maternal diet during gestation and lactation in the establishment of the offspring microbiome.	CTD or HFD cohorts were designated randomly. Microbiota: 16S rDNA of bacteria was amplified and 454 sequencing.	HFD early exposure: ↑ levels of *Bacteroidete*s and absence of *Spirochetes.* These shifts were primarily due to a great decrease of *Treponema* and increased relative abundance of *Prevotella.* HFD during pregnancy or post birth results in dysbiosis of the neonatal intestinal microbiome and partially corrected by low-fat, control diet after weaning.
Gibson et al. (2015)	2015	CC (Rats model)	9 offspring per diet experiment.	How *in utero* exposure to fish oil, rich in n-3 PUFA, caused abnormal intestinal reparative responses to mucosal injury through differences in intestinal microbiota.	Gut microbes, using qPCR, was compared between rat pups born to dams fed either n-6 PUFA, n-3 PUFA or control feed.	Diets rich in either n-3 PUFA or n-6 PUFA ↓ bacterial density, ↓ Firmicutes/Bacteroidetes ratio and ↓ several dominant microbes in the offspring’s gut. Excess n-3 PUFA was associated with blooms of potentially pathogenic microbes.
Chu et al. (2016b)	2016	PC	163 mother- infant pairs	To ascertain whether a maternal HFD similarly alters the neonatal and infant gut microbiome in early life.	Stool and meconium from neonates at delivery and at 6 weeks of age. Microbiota: 16S rRNA gene sequencing and analysis.	A notable relative depletion of *Bacteroides* in the neonates exposed to a maternal HFD until 6 weeks of age.
Selma-Royo et al. (2021)	2021	Nested cross-sectional study	73 mother- infant pairs	To ascertain the possible associations between maternal dietary intake during pregnancy and neonatal microbiota at birth.	Maternal-neonatal microbiota profiling at birth was assessed by 16S rRNA gene sequencing. FFQ to collet maternal nutrient intake during pregnancy	Maternal diet is associated with both maternal and neonatal microbiota at the time of birth, in a delivery mode-dependent manner. Maternal fat intake (↑SFA & MUFA) → ↑ Firmicutes in neonatal microbiota. Fibre, proteins from vegetable sources and vitamins → ↓ Firmicutes in neonatal microbiota.
Vegetarian diet						
Lundgren et al. (2018)	2018	PC	145 mother-infants pairs	To examine the association of maternal diet during pregnancy with the infant gut microbiome 6 weeks post-delivery.	Maternal prenatal diet: FFQ. Infant gut microbiota: Sequencing of the 16S rRNA V4-V5 hypervariable region and stratified analyses by delivery mode.	↑ fruit intake →↑ probability of high *Streptococcus/ Clostridium* group among infants born vaginally. Maternal dairy intake: →↑ probability of *Clostridium* in infants born by C-sections. Additional associations between maternal diet and infant intestinal microbes in both delivery mode strata.
Fan et al. (2021)	2021	PC	39 mother-infants pairs	To study the effects of high or low fruit and vegetable gestational intake on the infant microbiome.	Mothers: Complete 3-day dietary record and received postpartum follow-up. Infant gut microbiota: The 16S rRNA gene sequence at 2 months.	Infant gut microbiome clustered differently for high and low maternal fruit and vegetable consumption. ↑vegetable and fruit intake- ↑ *Propionibacteriales, Propionibacteriaceae, Cutibacterium, Tannerellaceae, Parabacteroides,* and *Lactococcus*.
Probiotics food consumption						
Bisanz et al. (2015)	2015	CC	56 pregnant women in Tanzania (26 received yogurt daily; 30 untreated during the last two trimesters and for 1 month after birth)	To study if maternal consumption of a Moringa-supplemented probiotic yogurt during pregnancy had an impact on gestation and health parameters in newborns.	Infant gut microbiota: analyzed using 16S rRNA gene sequencing. Maternal diet: dietary recalls were recorded.	Yogurt → ↑relative abundance of *Bifidobacterium +* ↓ *Enterobacteriaceae* in the newborn feces.
Tunay and Kök (2022)	2022	CC	30 lactating mothers (15 consumed kefir for 30 days; 15 volunteers (placebo group).	To investigate the vertical transmission of *Lactobacillus kefiranofaciens* subsp. *kefiranofaciens*, *Lentilactobacillus kefiri, Lentilactobacillus parakefiri* from mother to infant via consuming natural kefir.	Infant stool samples: DNA was characterised using qRT-PCR.	The mothers who consume the kefir for 30 days had indeed transfer of those unique bacteria of kefir in the human milk and infant stool.
Sweetened consumption						
Laforest-Lapointe et al. (2021)	2020	PC	100 (based on maternal ASB consumption: 50 non-consumers and 50 daily consumers).	To study if maternal consumption of ASB during pregnancy is associated with modifications of infant gut bacterial community composition.	Infant microbiota (at 3-4 months and 1 year): 16S rRNA gene sequencing.	4 microbiome clusters were identified: from immature (Cluster 1) to mature (Cluster 4) and two deviated from this trajectory (Clusters 2 and 3). Maternal ASB consumption was associated with community-level shifts in infant gut bacterial taxonomy structure and depletion of several *Bacteroides sp*. in Cluster 2.
Alcohol consumption						
Wang et al. (2021)	2021	PC	29 mother–child dyads were enrolled in central China (10 alcohol consumption during pregnancy).	To explore the effect of alcohol consumption and maternal diet during pregnancy on maternal and infant’s gut microbiota.	Fecal samples of newborns: The V3-V4 regions of 16S rRNA sequences were analyzed. A self-administrated questionnaire about simple diet frequency in the past week was completed by mothers before childbirth.	Alcohol consumptio affect β-diversity) in newborns. ↑ alcohol consumption ↑ *Megamonas* in newborns. The diet was not associated with both maternal and infant’s gut microbiota.

PC: Prospective cohort study; CC: cases-controls study; RCT: ramdomized controlled trial; FFQ: food frequency questionaire; HMOs: human milk oligosacharides; HFD: high fat diet; SFA: saturated fatty acids; MUFA: monounsaturated fatty acids; PUFA: polyunsaturated fatty acids; HPLC/MS: high performance liquid chromatography-mass spectrometry; ASB: artificially sweetened beverages; EBFI: exclusive breast fed infants; FFI: formula fed infants.

##### 5.1.3.1. High fat diets

A high fat diet during pregnancy and its effects in offspring is one of the most studied patterns among researchers (Ma et al., 2014; Gohir et al., 2015; Chu et al., 2016b; Mann et al., 2018). Ma et al. (2014) concluded, in a primate model, that maternal consumption of high fat diet during pregnancy or post birth results in dysbiosis of the neonatal intestinal microbiome. They observed the dominance of *Bacteroidetes* and an increase in the abundance of *Prevotella* in the offspring’s gut (Ma et al., 2014). Chu et al. (2016b) carried out a study in humans and demonstrated that independently of the maternal body mass index, a high fat maternal diet reduced Bacteroides levels in the neonatal gut microbiome.

Some authors have focused their research on how intake or exposure to certain specific fats such as fish oil, polyunsaturated fatty acid (PUFA) and monounsaturated fatty acids (MUFA), or vitamins influence the microbiota. For example, Gibson et al. (2015) compared the intestinal microbiota among offspring of rats born to mothers fed either n-6 (PUFA), n-3 PUFA or control feed; the authors concluded that there is a general reduction in microbial richness due to maternal exposure to a diet rich in PUFA. It was also observed an increase in the level of Bacteroidetes in the gut microbiota of offspring born to mothers who had consumed a diet rich in fish oil and also a reduction of *Enterobacteriaceae* and *Bifidobacteria spp.* and an abundance of taxa of opportunistic pathogens like *Bacteroides fragilis*, *Bilophila wadsworthia* and *Enterococcus faecium* when compared to the control group (Gibson et al., 2015). However, Urwin et al. (2014) did not found significant effect on maternal or infant gut microbiota based on the consumption of salmon, rich in n-3 and n-6 PUFA. When the authors took into account the mode of feeding, they observed that formula-fed infants in the salmon group were associated with lower bacterial counts of the *Atopobium* cluster compared to controls, though this was not observed in breastfed infants (BFI).

Mandal et al. (2016) studied, in 60 women in the second trimester of pregnancy, the relationships between the intake of macro and micro nutrients from diet and their gut microbiota through the use of food frequency questionnaires and observed, after delivery, taxonomic differences. They concluded that some of the most important modulators of the mother’s gut microbiota are fats and fats soluble vitamins. Specifically, they observed that a higher dietary intake of fat-soluble vitamins, especially vitamin D, were associated with reduced microbial alpha diversity. In addition, vitamin D, mono unsaturated fat, cholesterol and retinol were associated with higher levels of Proteobacteria, a phylum known to be associated with multiple pathogens and to have pro-inflammatory properties. On the other hand, the authors observed that saturated fats, vitamin E and proteins were associated with a relative reduction in Proteobacteria levels (Mandal et al., 2016).

Newly, Selma-Royo et al. (2021), in a nested cross-sectional study (MAMI cohort) observed that intake of saturated (SFA) and MUFA, is associated with both maternal and neonatal microbiota at the time of birth, in a delivery mode-dependent manner. Members of Firmicutes in neonatal gut microbiota were positively associated with a high fat intake, more specifically SFA and MUFA, during pregnancy, and negatively associated with a high consumption of fiber, proteins from vegetable sources, and vitamins by the mother.

##### 5.1.3.2. Very low-calorie ketogenic diet

The very low-calorie ketogenic diet (VLCKD) is a type of diet that is gaining much interest as a therapeutic approach to many diseases such as epilepsies (Lim et al., 2022; Zambrano et al., 2023). However, little is known about its effects during pregnancy on the mother’s or offspring’s gut microbiota and health. A few researchers have investigated the VLCKD, its possible influence on the gut microbiota of children and its therapeutic implications in asthma (Alsharairi, 2020b), obesity (Alsharairi, 2021) and inflammatory bowel diseases (Alsharairi, 2022) but not in pregnant women or infants.

The VLCKD is characterized by a low carbohydrate, moderate protein, and high fat diet (Lim et al., 2022). This low carbohydrate intake changes the energy source from glucose to ketone bodies (acetoacetate and β-hydroxybutyrate) allowing to achieve nutritional ketosis. The VLCKD includes sources of dietary fiber, fats (high in PUFA, moderate in MUFA and low in saturated fats) and plant-based protein, which may influence the development of SCFA-producing bacteria, and therefore, lead to an anti-inflammatory state. Furthermore, ketone bodies play an important role in regulating gut homeostasis and gene expression through epigenetic modifications similar to butyrate (Alsharairi, 2020b, 2022).

Despite all the potential benefits of the VLCKD, it would be premature to recommend this type of diet during pregnancy. Pregnancy is a period when nutritional requirements change according to the stage and where a shortage of macro- or micronutrients can trigger adverse health effects in both mother and offspring. At present, there is a paucity of high-quality clinical trials and prospective cohort studies to monitor changes in maternal gut microbiota composition during pregnancy and lactation following the VLCKD and their potential long-term effects on maternal and offspring health.

##### 5.1.3.3. Vegetarian diet

Nowadays, plant-based and vegetarian eating pattern are very extended. This type of diet is related to lower risk of obesity, cardiovascular disease, cerebrovascular disease, diabetes mellitus, and chronic kidney disease in adults (Miko et al., 2022).

In relation to its role in the gut microbiota, the vegetarian diet, due to fiber content, could promote beneficial changes such as an increased synthesis of SCFA.

To date, there is limited information on how a vegetarian diet during gestation may affect the gut microbiota of the newborn. There are two studies that investigated the role of a vegetarian or fruit-rich diet during gestation on the gut microbiota of the infant and in both cases different results were observed. Lundgren et al. (2018) found a negative association between maternal diets high in fruits and vegetables and the beneficial microbe *Bifidobacterium* in vaginally born infants. On the other hand, Fan et al. (2021) studied the effects of high or low fruit and vegetable gestational intake on the infant gut microbiota and did not observe any change in the alpha diversity but the authors did observe a variation in the infant’s microbiome. Specifically, the counts of *Propionibacteriales, Propionibacteriaceae, Cutibacterium, Tannerellaceae, Parabacteroides*, and *Lactococcus* were higher in the microbiome of infants with high maternal vegetable and fruit consumption.

##### 5.1.3.4. Probiotics food consumption

One of the nutritional recommendations for pregnant women is to increase the consumption of dairy products, including yogurt. Yogurt is a food produced by the bacterial fermentation of cow’s milk and it is considered a probiotic food since *Lactobacillus bulgaricus* and *Streptococcus thermophilus* are living in its preparation. Bisanz et al. (2015) assessed the influence of maternal consumption of probiotic yogurt with *Lactobacillus rhamnosus* GR-1 supplemented with Moringa plant on the infant microbiome and found an association between the maternal consumption of yogurt and an increase in the relative abundance of *Bifidobacterium* and a decrease in the relative abundance of *Enterobacteriaceae*.

Another food that is being consumed is Kefir. Kefir is a unique fermented dairy product that is produced by mixing lactic acid bacteria, acetic acid bacteria and yeast. Tunay and Kök (2022) investigated the possible effect of kefir consumption during gestation on the intestinal microbiota of the newborn and observed that had indeed transferred unique bacteria of kefir (*Lactobacillus kefiranofaciens* subsp. *kefiranofaciens, Lentilactobacillus kefiri* and *Lentilactobacillus parakefiri*) to the infant gut.

##### 5.1.3.5. Sweetened consumption

The consumption of sweeteners is very popular among the population. Nowadays, there is a high concern, about the negative impact that high sugar intake has on health and maybe this is one of the reasons why the use of sweetener, such us aspartame, sucralose or acesulfame-K, it has become very popular. One of the main foods that include sweeteners in their list of ingredients are sweetened beverages.

Laforest-Lapointe et al. (2021) study if maternal consumption of artificially sweetened beverages during pregnancy is associated with modifications of infant gut bacterial community composition at 3-4 months and 1 year of live and identified 4 microbiome clusters. From immature (Cluster 1) to mature (Cluster 4) and two deviated from this trajectory (Clusters 2 and 3). Alterations in infant gut bacterial taxonomy structure at the community level were associated with maternal artificially sweetened beverages consumption. A reduction of several *Bacteroides sp.* in Cluster 2 was also observed. The authors concluded that regular intake of artificial sweetened beverages during gestation resulted in a higher BMI of 1 year old infants, suggesting that infant gut microbiota and their metabolites could be involved.

##### 5.1.3.6. Alcohol consumption

Today there is no doubt about the negative effects of alcohol consumption during pregnancy, both for mothers and newborns. The effect of alcohol consumption during pregnancy was reviewed recently by Kumbhare et al. (2019), concluding that it is associated with various disorders such as hepatic diseases in the newborn and premature delivery.

Moreover, the initial intestinal colonization of the infant can be affected by such changes, making it more predisposed to infections and diseases later in life. However, how alcohol consumption during pregnancy affect infant gut microbiota are rarely investigated.

Wang et al. (2021) examined the relationship of maternal diet and alcohol consumption during pregnancy with the infant gut microbiota in Chinese mother-infant dyads. Alcohol intake leaded to a significantly lower abundance in *Faecalibacterium* genus compared with those without alcohol drinking. These bacteria have been shown an intestinal anti-inflammatory effect. Besides, *Megamonas* genus was abundant in infant exposed to alcohol consumption mothers. Nonalcoholic fatty liver disease, the most chronic liver disease in children and adolescents, is positively related to *Megamonas* in infant gut. However, it has been taken into account that more researches are needed to explore these relationships in a larger birth cohort.

In general, all these studies emphasize that maternal diet during pregnancy is a very influential factor in the development of the intestinal microbiota of both the mother and the newborn, and therefore, a key in the health of the infant during the first years of life.

#### 5.1.4. Gestational Age

One of the most important perinatal factors in the establishment of the infant gut microbiota is the gestational age. The World Health Organization (WHO; UNICEF and WHO, 2010) defined preterm births as those occurring before 37 completed weeks of gestation and usually have very low birth weight. During the pregnancy period, microbial diversity within the vaginal microbiota decreases, while members of *Lactobacillus* species increase, potentially reinforcing their protective function. However, infections caused by bacteria, viruses, and fungi during the pregnancy period have been considered a cause of intrauterine growth restriction and preterm birth. Certain infectious agents can reach the amniotic fluid, establishing an intra-amniotic infection and initiating an inflammatory response at the maternal and fetal tissues, which promotes pre-labor rupture of membranes and preterm birth (Ruiz et al., 2016). Bacterial communities characterized by high levels of *Atopobium*, *Gardnerella* and *Ureaplasma* as well as lower levels of *Lactobacillus* spp. or a higher presence of *Candida albicans* have been found to be correlated with premature deliveries (Ruiz et al., 2016).

Preterm infants (PTI) have to overcome serious health challenges. In most cases, and depending on the degree of prematurity, PTI have a degree of immaturity in the digestive system and respiratory, immune and neurological problems. Because of that, hospitalization is necessary for the application of treatments such as intensive use of antibiotics and other medications, and/or even the use of artificial respiration and feeding such as sterile parenteral nutrition (Henderickx et al., 2019). Also, in most cases the births of premature infants are usually by CS and therefore they never come in contact with the mother’s vaginal microbiome. All these factors can interfere in the process of colonization and the correct development of the gut microbiota, resulting in an unusual establishment and a different or deviating composition with increased colonization by pathogenic microorganisms.

The pattern of colonization in PTI is characterized by a reduction in bacterial diversity (Rougé et al., 2010; Moles et al., 2013; Henderickx et al., 2019). Several authors have compared the gut microbial colonization in preterm and full-term infants (FTI) and reporting that PTI showed a reduced level of strict anaerobes such as *Bifidobacterium*, *Bacteroides*, and *Atopobium*, and high levels of facultative anaerobes like *Enterobacter*, *Lactobacillus* and *Enterococcus*. It was also observed an increased abundance of *Enterobacteriaceae* family and *Staphylococcus*, and a higher colonization by pathogens, such as *Klebsiella* (Mshvildadze et al., 2010; Rougé et al., 2010; Arboleya et al., 2012, 2015). Additionally, Hill et al. (2017) in the INFANTMET cohort (using 16S rRNA amplicon Illumina sequencing and bacteriological culture) observed that at the phylum level Proteobacteria and Firmicutes dominates PTI when compared to FTI. As a result of the alterations in the composition of the intestinal microbiota, differences have also been observed in the main microbial metabolites, the SCFA, whose concentration in feces is higher in FTI than in premature neonates (Arboleya et al., 2012).

As it has been mentioned previously, the infant’s microbiota is not only composed by bacteria, but also of non-pathogenic viruses or archaea. In this respect, Rao et al. (2021) studying the forces that shape the dynamics of microbiome assembly in preterm neonates, observed that there was an inverse correlation between bacterial and fungal loads in the infant gut. Specifically, the authors found that interactions between different kingdoms may influence assembly. For example, *Candida albicans,* a fungal species, inhibited multiple dominant genera of gut bacteria.

#### 5.1.5. Mode of delivery

The mode of delivery has been recognized as an important driver of the early gut microbiota composition and have a major impact on the type of microbiota acquired during birth by the newborn. Several studies have shown that human neonatal microbiota across all body habitats (skin, oral, nasopharyngeal, and gut) is influenced by their mode of delivery (Penders et al., 2006; Biasucci et al., 2008; Dominguez-Bello et al., 2010). During the labor and immediately after birth, microbes from the mother and surrounding environment colonize the gastrointestinal tract of the infant leading to the development of a dense complex microbiota, being the first major exposure of the neonate to microbes (Munyaka et al., 2014; Korpela, 2021).Vaginal delivery infants (VDI) come into contact with the maternal vaginal and fecal microbiota, which results in neonatal gut colonization by vagina-associated microbes such as *Lactobacillus*, *Escherichia*, *Bacteroides*, *Bifidobacterium*, *Streptococcus* spp. and *Prevotella* (Penders et al., 2006; Dominguez-Bello et al., 2010; Fallani et al., 2010; Azad et al., 2013; Laursen et al., 2015). In contrast, children born by caesarean section (CS) are also exposed to their mother’s microbiota, but initial exposure is most likely to non-maternally derived environmental isolates from equipment, air, and other infants, with the nursing staff serving as vectors for transference (*Staphylococcus*, *Corynebacterium*, *Propionibacterium*; Biasucci et al., 2008; Dominguez-Bello et al., 2010; Munyaka et al., 2014).

According to the numbers provided by the WHO, in Spain more than 25 % of births are performed by CS and globally, the number of CS has grown from 12 % in the year 2000 to 21 % in 2015 over the world (Betran et al., 2016). The early colonization patterns of CSDI differ greatly from children born vaginally and being less diverse (Biasucci et al., 2008; Dominguez-Bello et al., 2010; Matamoros et al., 2013; Arrieta et al., 2014; Jakobsson et al., 2014). In particular, it was demonstrated that CSDI had minor amounts of *Bifidobacteria* (Dominguez-Bello et al., 2010; Azad et al., 2013), *Escherichia -Shigella*, and absence of *Bacteroides* (Fallani et al., 2010; Jakobsson et al., 2014) while were enriched with *Clostridium* (cluster I), *Clostridium difficile*, *Staphylococcus* species (Penders et al., 2006) and higher amounts of *Klebsiella* (Dogra et al., 2015). In contrast, VDI were characterized by *Bifidobacteria* (Hansen et al., 2015), predominantly *B. longum* and *B. catenulatum* species (Biasucci et al., 2008; Dogra et al., 2015) and vaginal-related microbes such as *Lactobacillus*, *Prevotella*, and *Sneathia* (Penders et al., 2006; Biasucci et al., 2008; Dominguez-Bello et al., 2010).

Also, Jakobsson et al. (2014) showed that the gut microbiota of CSDI at 24 months of age is less diverse than those delivered vaginally. The authors hypothesize that this drop in diversity may be due to delayed colonization of the gut by Bacteroidetes until 1 year of age. These differences between vaginally and CSDI gradually decrease, but remain more heterogeneous than VDI up to 12 months of life. In one recent research, focused on the long-term impact of delivery mode on gut microbiota development over the first 4 years of life, it has been found that *Lachnospiraceae* was dominant in CSDI while *Parabacteroides* was found to be more unique to VDI at one and 2 years of age; however, by the fourth year, *Lachnospiraceae* and other *Clostridium spp.* became more dominant in these VDI (Fouhy et al., 2019).

The differences observed in the microbiota between VDI and CSDI have been associated with the protective effect of vaginally delivered labor, particularly since it has been suggested that CS has long-term health implications. In fact, CS has been associated with an increased risk of immune disorders such as asthma (Ege et al., 2011; Roduit et al., 2009; Thavagnanam et al., 2008), allergy (Bager et al., 2008), type 1 diabetes (Cardwell et al., 2008), as well as an increased risk of overweight (Tun et al., 2018), a higher risk of development of inflammatory bowel disease (Bager et al., 2012) or an enhanced risk for developing celiac condition (Koletzko et al., 2018). Moreover, different modes of delivery affected the antibiotic resistance gene prevalence, that might influence the infant’s health later in life (Shi et al., 2018). The cause of this could be an inadequate development of the immune system since in several studies it has been observed that CSDI have a much lower level of Th1-related chemokines in their blood, which may be translated into less protection (Munyaka et al., 2014).

On the other hand, there are some authors who have not observed any association between CSDI and type 1 diabetes or the development of celiac disease (Sevelsted et al., 2015; Koletzko et al., 2018). Despite these studies, it can be concluded that the relevance of early gut microbiota in the maturation and development of the host’s immune system is supported by the finding that the mode of delivery influences the health status through adulthood, while the effects on gut microbiota composition decrease after the first year of life (Milani et al., 2017). The most accepted explanation for the association between mode of deliver and the development of diseases is the gut microbial dysbiosis, but it is necessary to carry out more research to adequately support this hypothesis.

### 5.2. Postnatal factors

Some of the most important postnatal factors, that may influence the gut microbiota composition of a growing neonate are the mode of infant feeding, the introduction to solid food, environment and lifestyle, the use of antibiotics and host genetics.

#### 5.2.1. Infant feeding during the first months of life

##### 5.2.1.1. Breast milk vs. infant formula

Mostly, breast milk (BM) is the first food to which the newborn is exposed, and it is during breastfeeding when one of the most important links between the mother and the newborn is formed. Although nowadays infant formulas (IF) are products increasingly similar to BM, large differences continue to be observed. This is because BM has a very complex composition mainly due to the presence of various growth factors and enzymes, and also this composition changes over time (colostrum, transitional milk and mature milk; Scholtens et al., 2009; Bezirtzoglou et al., 2011; Cabrera-Rubio et al., 2012; Guaraldi and Salvatori, 2012; Andreas et al., 2015). It has been reported, that BM and more specifically its bioactive compounds, such as HMO, probiotics polyamines, lactoferrin, nucleotides and whey proteins affect different biological processes, intervening in the optimal physical and intellectual development of the child, as well as in the reduction of the incidence of future diseases (Turfkruyer and Verhasselt, 2015; Oddy, 2017; Moossavi et al., 2018), as Kumbhare et al. (2019) and Porro et al. (2023) reviewed. For this reason, WHO recommends exclusive breastfeeding of infants up to 6 months due to the considerable potential to improve child health and wellbeing (UNICEF and WHO, 2010).

The differences in the gut microbial composition between the infants who has been fed with BM and those who have been fed with IF are well documented (O’Sullivan et al., 2015; van den Elsen et al., 2019) and Table 2 summarizes the main studies carried out to understand how diet and nutrition may have an influence on influent the infant gut microbiota during the first months of life.

**Table 2:** Diet and nutrition during first months of life: description of the studies.

References	Year	Type of study	Study population	Objective	Methods	Summary of major finding
Breast milk vs infant formula						
Harmsen et al. (2000)	2000	CC	12 newborn infants (6 BFI and 6 FFI).	Novel molecular identification methods were used to verify the data obtained by traditional culture methods and to validate the culture independent FISH technique.	6 fecal samples obtained during the first 20 days of life. Microbial compositions analysis: culturing on specific media and by FISH.	In all BFI, *Bifidobacteria* become dominant and the minor components were mainly *Lactobacilli* and *Streptococci.* FFI similar amounts of *Bacteroides* and *Bifidobacteria* (approximately 40%) and often contained *Staphylococci, Escherichia coli,* and Clostridia.
Penders et al. (2006)	2006	PC	1,032 infants at 1 month of age.	To examine the contribution of a broad range of external factors to the gut microbiotic composition in early infancy.	qPCR assays for the enumeration of *Bifidobacteria, Escherichia coli, Clostridium difficile, Bacteroides fragilis* group, *Lactobacilli,* and total bacterial counts.	Exclusively FFI ↑ *E coli, C difficile, Bacteroides* and *Lactobacilli*, compared with BFI.
Fallani et al. (2010)	2010	PC	606 infants (age 6 weeks).	To assess the impact of geographic area, mode of delivery, feeding method, and antibiotic treatment on the fecal microbiota of infants from 5 European countries with different lifestyle characteristics.	Fecal samples analyzed by FISH + flow cytometry using a panel of 10 rRNA targeted group- and species-specific oligonucleotide probes. Factors collected with questionnaires.	*Bifidobacteria* dominated the microbiota of BFI, whereas FFI ↑ proportions of *Bacteroides* and members of the *Clostridium coccoides* and *Lactobacillus* sp.
Bezirtzoglou et al. (2011)	2011	CC	12 healthy vaginally delivered newborn infants (6 BFI and 6 FFI)	To evaluate the microbiota in newborns vaginally delivery under different types of feeding using molecular tools.	FISH: molecular evaluation of the microbiota in vaginaly delivery newborns. Eleven probes/probe combinations for specific groups of faecal bacteria.	BFI: ↑ numbers of *Bifidobacterium* compared to FFI. After FFI: microbiota more diverse and ↑*Atopobium*, ↓*Bifidobacterium* followed by ↑ *Bacteroides.*
Azad et al. (2016)	2013	PC	198 healthy term infants from the CHILD Study.	To determine the impact of IPA on infant gut microbiota, and to explore whether breastfeeding modifies these effects.	Maternal information: hospital records and breastfeeding was reported by mothers. Infant gut microbiota: Illumina 16S rRNA sequencing of faecal samples at 3 and 12 months.	Microbiota differences were especially evident following IPA with emergency CS, with some changes (increased *Clostridiales* and decreased *Bacteroidaceae*) persisting to 12 months, particularly in non-BFI.
Ardissone et al. (2014)	2015	PC	49 EBF newborns (who were also vaginally delivered and ceased BF between 4 and 12 months).	To characterized the gut microbiome during the first year of life and assess the impact of mode of delivery and feeding on its establishment.	Study relative abundances of all MetaOTUs and 64 MetaOTUs (27 of which were novel species) were selected as markers for gut microbiota age.	At 1 week: type of feding not affect the newborn microbiome. At 4 months: EBFI ↑ levels of *L. johnsonii/L.gasseri, L. paracasei/L. casei,* and *B. longum* (probiotics properties). FFI ↑ levels of *Clostridium difficile*, *Granulicatella adiacens, Citrobacter* spp.*, Enterobacter cloacae, Bilophila wadsworthia.* FFI enriched in *B. adolescentis* consistent with its positive to negative correlation with *B. longum* from newborn to 4 months.
Martin et al. (2016)	2016	PC	108 healthy neonates until the first half year of life.	To study the composition of the microbiota in early life and the evolution of the microbial community during the first 6 months of life.	Microbial composition: measuring 33 different bacterial taxa by qPCR/RT qPCR. Information regarding gender, place and mode of birth, presence of siblings or pets; feeding pattern and antibiotic use was collected by using questionnaires.	Type of feeding influence the colonization of *Bifidobacterium, Lactobacillus* and *Bacteroides* at species level and over time. *B. animalis subsp. lactis* presence was found to be dependent solely on the type of feeding, indicating that it might not be a common infant gut inhabitant.
Ruiz et al. (2016)	2017	CC	163 infants (at 3–5 months of age)	To investigate differences in fecal SCFAs and intermediate metabolites in infants according to breastfeeding status	Mothers reported infant feeding practices using standardized questionnaires administered at 3, 6, and 12 months postpartum. Samples were analyzed using NMR spectroscopy.	EBFI: ↓ total SCFAs and ↑ lactate. ↑ relative proportion of acetate. EBFI were four times more likely to have a higher proportion of acetate relative to other SCFAs in their gut. This association was independent of birth mode, IPA, infant sex, age, recruitment site, and maternal BMI or socioeconomic status.
Complementary feeding						
Bergström et al. (2014)	2011	PC	605 infants	To determine the impact of weaning on the faecal microbiota composition of infants from five European countries which have different lifestyle characteristics and infant feeding practices.	Faecal samples were collected approximately 4 weeks after the introduction of first solid foods and before weaning (6 weeks of age). Gut microbiota: FISH and flow cytometry using a panel of 10 rRNA targeted group- and species-specific oligonucleotide probes.	"After weaning →*Bifidobacterium*, *Clostridium* coccoides group and Bacteroides were predominant. The initial feeding method influenced the Clostridium *leptum* group and *Clostridium* difficile + *Clostridium* perfringens species. BFI: *Bifidobacteria* dominated the faecal microbiota. FFI: ↑proportions *Bacteroides* and the *C. coccoides* group. "
Laursen et al. (2016)	2014	PC	330 infants (at 9, 18, and 36 months after birth)	To determine nutritional parameters and measures of growth and body composition in relation to the observed development in microbiota composition.	Use of qPCR targeting 31 selected bacterial 16S rRNA gene targets representing different phylogenetic levels.	Weaning period (9–18 months): replacement of a microbiota characterized by *Lactobacilli*, *Bifidobacteria*, and *Enterobacteriaceae* with a microbiota dominated by *Clostridium* spp. and *Bacteroides* spp. The composition of the microbiota was most pronouncedly influenced by the time of cessation of breastfeeding.
Thompson et al. (2015)	2015	PC	49 stool samples collected of 9 infants (5 male, 4 female)	To examine differences in the intestinal microbiome between four feeding groups; EBFI before introduction of solid foods, non-EBFI before introduction of solid foods, EBFI after introduction of solid foods (EBFI+S), and non-EBFI after introduction of solid foods (non-EBFI+S).	Bacterial 16S rRNA amplicon sequencing was performed.	"EBFI: ↑ proportions of *Bifidobacterium*, ↓ *Bacteroidetes* and *Clostridiales* than non-EBFI. Introduction of solid foods had a marginal impact on the microbiome of EBFI. In contrast, over 200 bacterial gene categories were overrepresented in non-EBFI+S compared to non-EBFI."
Sevelsted et al. (2015)	2016	PC	227 infants [born either of a random sample of healthy mothers (*n* = 114), or of obese mothers (*n* = 113)]	To elucidate the impact of (i) maternal obesity and (ii) dietary factors on infant gut microbiota development, associations between specific features of the gut microbiota and dietary factors were investigated with a focus on breastfeeding and complementary diet composition.	"Infant gut microbiotas: 16S rRNA amplicon sequencing. Gut microbiota data were compared to breastfeeding patterns. Infant diet: individual dietary recordings."	Infant gut microbial composition and alpha diversity were thus strongly affected by introduction of family foods with high protein and fiber contents. Specifically, intake of meats, cheeses, and Danish rye bread, rich in protein and fiber, were associated with increased alpha diversity.
Homann et al. (2021)	2021	PC	24 healthy, full-term infants from the Baby, Food & Mi and LucKi-Gut cohort	To evaluate the relationship between nutritional choices at the time of introduction to solid foods and gut bacterial dynamics in a cohort of full-term, vaginally born, and healthy infants.	"Infant diet: Caregivers provided food diaries. Stool samples: one sample before the introduction of solids, and multiple samples were collected after the introduction of solids. Infant gut microbiota: 16S rRNA amplicon sequencing"	"↑ dietary diversity → ↑ Microbial richness + ↑ diversity ↑ daily dietary diversity ↑ Bifidobacterial taxa ↓ Veillonella"

EBFI: exclusive breast fed infants; FFI: formula fed infants; MFI: mix fed infants; BFI: breast fed infants; MCP: methyl-accepting chemotaxis proteins; PC: prospective cohort study; CC: cases-controls study; SCFA: short chain fatty acids; qPCR: quantitative polimerase chain reaction; NMR spectroscopy: nuclear magnetic resonance spectroscopy; FISH: fluorescence in situ hybridisation; IPA: maternal intrapartum antibiotic prophylaxis.

The gut microbiome in formula fed infants exhibits higher diversity than that of BFI because the latter are exposed to different carbohydrates, bacteria, and nutrients, causing different microbial colonization patterns of the gut. In this context, different publications have reported that stools of BFI contain higher levels of *Bifidobacterium* species (phyla Actinobacteria), being the most prevalent *B. breve*, *B. longum*, *B. dentium*, *B. infantis* and *B. pseudocatenulatum* (Harmsen et al., 2000; Bäckhed et al., 2015). Also, BFI’s stools contain higher levels of *Lactobacillus* and lower levels of potential pathogens than those infants feeding with IF. Infants exclusively feed with IF were more often colonized by *Staphylococci*, *Bacteroides*, *Clostridium* species, *Enterococci*, Enterobacteria, and the genus *Atopobium* (Penders et al., 2006; Fallani et al., 2010; Bezirtzoglou et al., 2011; Guaraldi and Salvatori, 2012; Gritz and Bhandari, 2015; Azad et al., 2016; Martin et al., 2016).

As mentioned earlier, a different pattern of colonization also implies differences in microbiota metabolism and therefore SCFA levels (Porro et al., 2023). Exclusive breastfeeding has been associated with lower absolute concentrations of total SCFA, Acetate, Butyrate, Propionate, Valerate, Isobutyrate, and Isovalerate, and higher concentrations of lactate. Further, the relative proportion of acetate was higher with exclusive breastfeeding compared to infants feed with Infant formula (Bridgman et al., 2017).

##### 5.2.1.2. Complementary feeding

Although it is well established that early infant feeding has a major influence on the establishment of the gut microbiota, very little is understood about how the introduction of first solid food and the gradually replace of the milk-base diet influences the infant gut microbiota.

The introduction of solid foods is an important dietary event during infancy that causes profound shifts in the gut microbial composition towards a more adult-like state (Penders, 2017; Homann et al., 2021; Schwab, 2022). One of the reasons why this profound change occurs may be due to the progressive decrease in the consumption of breast milk during the complementary feeding stage and consequently of its components (HMOs and bacteria). In addition, with the introduction of solid foods, the diversity and complexity of dietary carbohydrates increases, leading to an increase in the abundance of carbohydrate-degrading bacteria in the months after switching to solid foods (Schwab, 2022). All this leads to a transition process in which the fermentative capacity increases after weaning, and which takes several months to complete. This increase in fermentative capacity produces substantial changes not only in the intestinal microbiota, but also in its metabolites, SCFAs. Specifically, butyric acid is the SCFA with most dramatic higher levels after the introduction of solid foods together with bacteria that produce it.

As Laursen et al. (2017), a large number of longitudinal studies have showed a significant change in microbial composition around the stage of introduction to solid foods and cessation of milk-base diet.

Bergström et al. (2014) carried out a large Danish cohort (“SKOT study”) to study the fecal microbiota of 330 infants at 9, 18 and 36 months of age. The authors observed, during the period characterized by transition from breastfeeding/formula feeding to family diet, that a complementary feeding induces replacement of a microbiota characterized by *Lactobacilli*, *Bifidobacteria*, and *Enterobacteriaceae* towards a microbiota dominated by *Clostridium* spp. and *Bacteroides spp.* On the other hand, since the microbiota is influenced by the age at which the novel foods are introduced, its composition was also pronouncedly influenced by the time of cessation of breastfeeding.

Fallani et al. (2011) in a European cohort formed by 531 infants from five different countries, observed similar results and demonstrate that, regardless of differences in geographic location, antibiotic use, delivery mode and milk feeding practices, there are consistent changes in the microbial composition of infants. Specifically, was observed a decrease in *Bifidobacteriaceae*, *Enterobacteriaceae* and *Clostridiaceae* while increasing in *Ruminococcaceae* and *Lachnospiraceae* species, starting at 6 weeks up to 4 weeks after introduction of solid food.

These results are in agreement with those found by Thompson et al. (2015) who also observed an increase in the diversity of the intestinal microbiota of infants both in exclusively BFI and non-exclusively BFI (n = 10) after introduction of solid foods. The authors observed a higher diversity and species richness in non-exclusively BFI compared with exclusively BFI. On the other hand, the pattern of exclusively BFI gut microbiota showed increased proportions of *Bifidobacterium* and a decrease in abundance of Bacteroidetes and Clostridiales with respect to non- exclusively BFI.

Laursen et al. (2016) observed a decrease in saccharolytic bacteria, such as members of *Bifidobacteria* genus, and an increase in *Lachnospiraceae*, which are associated with breast milk. These changes were correlated with a higher protein intake. On the other hand, ingestion of fiber was demonstrated to be associated with higher levels of *Prevotellacea*. Interestingly, two species that are absent or present at very low levels during early infancy, *Faecalibacterium prausnitzii* and *Akkermansia muciniphila*, increase in abundance to adult levels at 12 months and 24 months, respectively, (Milani et al., 2017). Both species are being widely studied because they are considered to promote a correct state of health in adulthood. Interestingly, the microbiota composition in African and European infants is very similar until the introduction of solid foods, indicating the dominant role of diet over other variables in shaping the microbial composition of the gut in early life (van den Elsen et al., 2019).

More recently, Homann et al. (2021) characterized the infant gut microbiota of 24 healthy, full-term infants, belonging from two different countries and concluded that introducing a high variety of first foods may increase alpha diversity and stabilize the gut microbiome early in life.

#### 5.2.2. Probiotic consumption in early life

Nowadays, the consumption of probiotics by infants during lactation and in the first years of life is very widespread as they are considered to have a collaborative role in solving different digestive problems. In this respect, Korpela et al. (2018) observed that probiotics had a strong overall impact on the infant gut microbiota composition, but the effect depended on the infant’s diet. Only breastfed infants showed the expected increase in *Bifidobacteria* and reduction in *Proteobacteria* and *Clostridia*. In the placebo group, both birth mode and antibiotic use were significantly associated with altered microbiota composition and function, particularly reduced *Bifidobacterium* abundance. In the probiotic group, the effects of antibiotics and birth mode were either completely eliminated or reduced. These indicate that it is possible to correct undesired changes in microbiota composition and function caused by antibiotic treatments or caesarean birth by supplementing infants with a probiotic mixture together with at least partial breastfeeding.

#### 5.2.3. Environment and lifestyle

Geographical location has been described as a relevant environmental factor that may have an impact on human microbiota. Different ethnogeography populations have distinct genetic backgrounds, dietary patterns, and cultural practices.

For example, De Filippo et al. (2010) compared the fecal microbiota of children from a rural African village of Burkina Faso and that of European urban infants, residents in Florence, Italy (EU). The reason to select these two populations was that the diet of the children from Burkina Faso is characterized by the consumption of grains, legumes and high-fiber diet of vegetables with the absence of processed food and furthermore, it represents a society similar to that of early human colony at the Neolithic era. EU children have a western diet, which is characterized by a high consumption of animal fats, sugars and a greater caloric intake. Gut microbiota from Burkina Faso children were dominated by Bacteroidetes and showed a depletion in Firmicutes population. In addition, a significantly high levels of SCFA were found.

Subsequently, further studies have continued investigating and comparing the differences between developing vs. developed countries including a large cohort of pediatric and adult samples from the Amazonas of Venezuela, rural Malawi and urban United States areas. *Bacteroides* predominated in the North American samples, whereas *Prevotella* predominated in Malawian and Venezuelan samples (Yatsunenko et al., 2012). As well as, there were many more differences in the samples from USA than between Malawian and Venezuelan microbiota mainly in children older than 3 years of age.

Three years later, Lin et al. (2013) observed the same *Prevotella*-*Bacteroides* split when the authors compared American children living in wealthy neighborhoods with children living in a Bangladesh slum. Another research comparing southeaster African and northern European infants reported a different bacterial composition, containing higher abundance of the *Prevotella*-*Bacteroides* group and the *Bifidobaterium* genus in African children (Grzeskowiak et al., 2012a).

In Spain, Echarri et al. (2011) evaluate the microbiota of 40 breastfed FTI from two different Spanish locations, one in the northern Atlantic coast and the other in the south-east Mediterranean coast, at different times (fecal samples were collected at 8, 30 and 90 days of life). The authors showed a high inter-individual variability on the levels of the different microbial groups. However, despite this variability their results showed statistically significant higher levels of *Bacteroides* and *Staphylococcus* (at 8 days of age) and lower levels of *Enterobacteriaceae* in infants from the Mediterranean coast than in infants from northern Spain. Similar levels of *Enterococcaceae*, Clostridia XIVa and IV clusters, *Atopobium*, *Bifidobacterium* and *Lactobacillus* were found between both groups at 90 days of life. When sampling times were not taken into account, lower counts of *Lactobacilli* and higher counts of *C. leptum group*, as well as significantly higher levels of *Bacteriodes* and *Staphylococcus* were observed in infants from the south of the country compared to those from the north coast.

These differences have also been observed in comparative studies of colonization patterns between different countries belonging to the same continent. For example, it has been reported that infants from Northern areas from Europe have higher levels of *Bifidobacterium* spp. and some *Clostridium spp.* and *Atopobium spp.*, while Southern European infants had a higher abundance of *Eubacteria*, *Lactobacillus*, and *Bacteroides* (Fallani et al., 2010). Also, other authors have showed significant differences between the gut microbiota of European infants from different countries (Grześkowiak et al., 2012b). For example, between German and Finnish infants, where German infants showed a higher abundance of the *Bacteroides-Prevotella* group and *Akkermansia muciniphila* while the proportion of *Bifidobacterium spp.* was higher in the Finnish infants (Grześkowiak et al., 2012b).

In addition to the geographical location, family structure and lifestyle may influence the model of colonization in infant gut microbiota and have also been describe as a relevant environmental factor (Rodríguez et al., 2015). Despite this, it is necessary to establish more decisive evidence about the effects of family structure, size, and birth order in the pattern of colonization (Fouhy et al., 2012).

Infants without siblings, who were recruited from the Child, Parent and Health: Lifestyle and Genetic Constitution (KOALA) Birth Cohort Study, had slightly lower numbers of Bifidobacteria, compared with infants with older siblings (Penders et al., 2006). These results agree with those reported by Adlerberth et al. (2007) in another research as part of the ALLERGYFLORA study. In this publication, infants with older siblings had lower proportions of Enterobacteria, other than *Escherichia coli*, as well as Clostridia, in the gut, but also a higher anaerobe/facultative anaerobe ratio. In a recent study performed with a Danish cohort, the presence of older siblings was shown to be associated with increased gut microbial diversity and richness during early childhood, while the presence of household pets had less-pronounced effects on the gut microbiota (Laursen et al., 2015).

Overall, geographical location (dietary patterns and lifestyle in a specific area and family structure (siblings) seems to affect gut microbiota colonization during early life, although more studies are needed to determined more accurately the factors that influence or contribute to a greater extent.

#### 5.2.4. Antibiotics during early life

Antibiotic usage in early life can also disrupt the normal pattern of colonization, affecting the growth of otherwise dominant bacterial phyla in the human gut (Reyman et al., 2022). These alterations can remain for long periods of time, spanning months and even years with partial or complete recovery (Kumbhare et al., 2019) and leading to a greater susceptibility to numerous diseases later in life (Johnson et al., 2005; Penders et al., 2006; Fallani et al., 2010; Chu et al., 2015; Rasmussen et al., 2018) such as asthma (Kozyrskyj et al., 2007; Metsälä et al., 2015).

Yassour et al. (2016) carried out a longitudinal study to investigate changes in the gut microbiome from 39 infants, half of whom received, during the first 3 years of life, multiple courses of antibiotics. The microbiota of antibiotic-treated children was less diverse in terms of both bacterial species and strains, with some species often dominated by single strains. In addition, they observed short-term composition changes between consecutive samples from children treated with antibiotics.

Additionally, Korpela et al. (2016) observed in 2–7-year-old Finnish children (n = 142; sampled at two time points) that the use of macrolides is associated with changes in microbial composition, reducing diversity and metabolism of the microbiota that also proves to be durable. Specifically, after 1 year of administration of finishing antibiotics treatment, the authors observed the restoration in the levels of *Bifidobacterium* and *Bacteroides* and antibiotic resistance and also concluded that Penicillins imprints a weaker mark on the microbiota in comparison with macrolides (Korpela et al., 2016). Finally, Reyman et al. (2022) observed, in 147 infants exposed to antibiotics in their first week of life, a decreased abundance of *Bifidobacterium spp.* and increased abundance of *Klebsiella* and *Enterococcus* spp. compared to controls. Also, regarding the type of antibiotic employed, the authors observed that Amoxicillin + Cefotaxime show the largest effect on microbial composition and also in anti-microbial resistance gene profile. In contrast, Penicillin + Gentamicin had minor effects (Reyman et al., 2022).

Another factor to consider is the duration of antibiotic treatment. About this theme, one study has investigated how short (≤ 3 days) or long treatments (≥ 5 days) may influence gut microbiota of infants in early life. Zwittink et al. (2018) observed a depletion in infant gut *Bifidobacterium* levels after a short treatment with antibiotics until the third week of life. In long treatments, this depletion in *Bifidobacterium* remains decreased up to 6 weeks postpartum. In both type of treatments with antibiotics, *Enterococcus* became the dominant genus of the microbial community. These results are in line with other authors (Tanaka et al., 2009; Martin et al., 2016; Fouhy et al., 2019). Specifically, Martin et al. (2016) observed a slightly depletion in total bacterial counts and also decreased levels of *Bifidobacterium* and *Staphylococcus* in infants who received antibiotics.

All these results suggest that the first moths of life are indeed a critical window where the infant gut microbiota could be influenced by the use of antibiotics and linking these changes with later diseases. However, more research is necessary to understand the impact of antibiotic treatment and their long-term effects. Moreover, issues related with the increasing number of pathogenic strains and their antibiotic resistance should be considered because of both are growing due to the ever-increasing use of antibiotic.

## 6. Conclusion

Although there is no consensus about prenatal colonization through microbial transmission between mother and child, there does exist a broad agreement on the main factors that may influence the transmission and subsequent colonization of the infant’s intestinal microbiota. Prenatal factors such as consumption of antibiotics and diet during pregnancy can affect the maternal gut microbiota and their metabolites, as well as breast milk composition, which in turn can affect infant colonization and even influence offspring’s future development and health.

Focusing on the diet during pregnancy, and summarizing the different bibliographical sources consulted, while this review has outlined significant advances in our understanding of the role of diet during pregnancy in the development of infant gut microbiota, large gaps still exist. Despite all the studies carried out, both in humans and in animal models, with the aim of determining the role of the consumption of macro and micro-nutrients during pregnancy and how it affects the infant gut microbiota, the wide differences in methodology and in the results observed do not allow us to obtain a clear conclusion. However, as other authors have indicated, alcohol consumption should be totally restricted during the gestation period, not due to possible changes in the intestinal microbiota of the child, but due to its adverse effects on mother’s and offspring’s health.

On the other hand, as previously mentioned, pregnancy is a stage with specific nutritional needs which also change throughout the gestation period. Modifications in the diet can lead to the restriction of certain macro or micronutrients, thus leading to altered health states of both the mother and the offspring. For example, ketogenic diets that restrict the consumption of carbohydrates, the main source of glucose, have not been studied in pregnant women and its short- and long-term effects are unknown.

The first moths of life are also a critical window where the infant gut microbiota could be influenced by postnatal factors, such as the use of antibiotics, environment and lifestyle agents and of course the infant diet, being linked these changes with later diseases. Regarding feeding during the first years of life, accumulating evidence from human observational and animal studies suggests that diet during the first months of life, and more specifically breastfeeding may influence the offspring microbiota in a good way. The breastfed infants seem to present higher levels of *Bifidobacterium* species and lower levels of *Bacteroides, Clostridium, Enterococcus, Enterobacteriaceae, Atopobium* and potential pathogen species, comparing with formula fed infants. Regarding the introduction of first solid foods and the gradually replacement of milk-based diets, a large number of longitudinal studies have shown a significant change in microbial composition, characterized by dominance of complex carbohydrate-degrading bacteria although additional studies with larger sample size would be required.

Finally, we consider it necessary to take into account two issues. On the one hand, the procedure of development and maturation of the intestinal microbiota is a dynamic and non-random process, in which both positive and negative interactions occur between the main microbial taxa. On the other hand, it is not a single factor, but a wide range of factors acting together, that can cause changes in this balance, thus altering the homeostasis of the intestinal microbiota and causing the so-called state of dysbiosis. All this makes it difficult for the scientific community to reach a solid conclusion about which factors are really influencing the colonization pattern and evolution of the intestinal microbiota of the infant and its repercussions on future health, generating the need to carry out more studies that can help to know and understand in more detail these relationships, taking into account more than one as a whole and study their effects in the short and long term.

## Author contributions

CM-G, MS-P, and GY-G: conceptualization and review and editing. CS-M: wrote the paper. All authors have read and agreed to the published version of the manuscript.

## Funding

This research was supported by the Spanish Ministry of Science, Innovation and Universities (grant number PID2019-106693RB-100/AEI/10.13039/501100011033). CS-M was funded by a predoctoral fellowship (FPU MECD 15/05809) awarded by The Ministry of Education, Culture as part of the Government of Spain.

## Conflict of interest

The authors declare that the research was conducted in the absence of any commercial or financial relationships that could be construed as a potential conflict of interest.

## Publisher’s note

All claims expressed in this article are solely those of the authors and do not necessarily represent those of their affiliated organizations, or those of the publisher, the editors and the reviewers. Any product that may be evaluated in this article, or claim that may be made by its manufacturer, is not guaranteed or endorsed by the publisher.

## Figures and Tables

**Table:** 

BFI	Breastfed infant
BM	Breastmilk
CS	Caesarean section
CSDI	Caesarean section delivered infant
FID	Flame ionization detector
FTI	Full term infant
HMO	Human milk oligosaccharides
IF	Infant formula
IPA	Intra partum antibiotic
MAC	Microbiota-accessible carbohydrates
MUFA	Monounsaturated fatty acids
PTI	Pre-term infant
PUFA	Polyunsaturated fatty acids
SCFA	Short chain fatty acids
VDI	Vaginally delivered infant
VLCKD	Very low caloric ketogenic diet
WHO	World Health Organization
